# Extensive Cellular Inflammation from Vaccination

**Published:** 1829

**Authors:** 


45. *Extensive cellular inflammation from Vaccination.*—We are indebted to Dr. Joseph N. McDowell, of this city, for the following memorandums of an interesting case which lately occurred in his practice, and fell under our own observation:

"In January last, says he, during the alarm excited by the small pox, I vaccinated Mrs. W——, about sixty years of age, possessing a good constitution, and, for that time of life, remarkably active. She was subject, however, to attacks of dysentery, which, at different periods of the year confined her for several days. The virus used, was such as had been successfully employed among all my patients, and was inserted in the arm in the usual manner by raising a small portion of skin with the point of the lancet and placing it underneath. Three days after the insertion of the infection, I returned to see whether it had taken effect; when I found her arm much inflamed, and learned that the night after her vaccination, she had been taken down with dysentery, which was attended from its commencement with fever. The unusual inflammation excited in her arm, she attributed to her having inadvertantly scratched it. At this time her dysentery still continued; her pulse was active, tongue furred, and she had great thirst. I urged her to be bled and take medicine. She consented to the former, but obstinately refused to do any thing more for several days, during which time the inflammation greatly increased, and her situation became alarming. I again visited her in company with Dr. Drake, when we prevailed upon her to submit to our directions. Her arm was, at this time, almost double the size of the other, and inflamed from near the shoulder to the ends of her fingers; and notwithstanding all our exertions, an extensive suppuration in the cellular membrane took place. The integuments were broken at four different places on the arm, and at two others on the fore arm, near the lower end of the radius. Between the elbow and the wrist they were entire; but from the extensive suppuration, they were separated from the muscles and hung loosely down when the arm was lifted up. During this stage of the disease, she was extremely irritable, and required to be kept constantly under the influence of opium, with tonics and aperients. The skin being widely separated from the muscles, there was danger of its being lost by sloughing. To prevent this I resorted to a bandage, extending from the ends of her fingers above the elbow, for the two fold purpose of reducing the œdema, and bringing the muscles and integuments into contact. Its effects were salutary, and it was, consequently, continued for some time. The orifices in the skin continued for a while as indolent running sores; but in the end were excited to healthy action, and healed up. From the excessive inflammation and suppuration she suffered a partial loss of power in the muscles of the hand; for a time was unable to move her fingers in any direction, and even at present is considerably infirm.

Cincinnati, June 25, 1829.

